# Linear stability analysis of magnetohydrodynamic duct flows with perfectly conducting walls

**DOI:** 10.1371/journal.pone.0186944

**Published:** 2017-10-27

**Authors:** Shuai Dong, Lishuai Liu, Xuemin Ye

**Affiliations:** Department of Power Engineering, North China Electric Power University, Baoding 071003, China; Worcester Polytechnic Institute, UNITED STATES

## Abstract

The stability of magnetohydrodynamic flow in a duct with perfectly conducting walls is investigated in the presence of a homogeneous and constant static magnetic field. The temporal growth and spatial distribution of perturbations are obtained by solving iteratively the direct and adjoint governing equations with respect of perturbations, based on nonmodal stability theory. The effect of the applied magnetic field, as well as the aspect ratio of the duct on the stability of the magnetohydrodynamic duct flow is taken into account. The computational results show that, weak jets appear near the sidewalls at a moderate magnetic field and the velocity of the jet increases with the increase of the intensity of the magnetic field. The duct flow is stable at either weak or strong magnetic field, but becomes unstable at moderate intensity magnetic field, and the stability is invariance with the aspect ratio of the duct. The instability of magnetohydrodynamic duct flow is related with the exponential growth of perturbations evolving on the fully developed jets. Transient growth of perturbations is also observed in the computation and the optimal perturbation is found to be in the form of streamwise vortices and localized within the sidewall layers. By contrast, the Hartmann layer perpendicular to the magnetic field is irrelevant to the stability issue of the magnetohydrodynamic duct flow.

## 1 Introduction

The problem of magnetohydrodynamic (MHD) flow through ducts has become important because of several industrial applications, such as design of the cooling blankets for nuclear fusion reactor, MHD pumps, measurement of blood flow, etc [1–3]. Although the Reynolds number is large, the flow considered is actually in transitional or weak turbulent state due to strong magnetic damping effect. The imposed magnetic field tends to suppress velocity gradient in the direction of the filed and give rise to electromagnetic (EM) boundary layer. The combination of magnetic damping and developing of EM boundary layers governs the transition to turbulence, as well as the properties of developed MHD turbulent flow [4]. Instability and turbulence could strongly affect the mass, heat and momentum transfer in MHD flows in practice. Unfortunately, we still know little about the mechanism of instability and transition in such kind of flows. There is rare data reported on this topic due to the difficulty of performing related experiments and measurements. The conducting fluid in practice is usually opaque, which makes it impossible to get a direct observation and record of the flow structures. One may want to resort to transparent electrolytes, but they usually have very low conductivity, which preclude their use in experiments. It is also very difficult to build a system to generate the demanded magnetic field, which is both strong and uniform over a large test area. There are still safety concerns due to oxidation and erosion of conducting fluids, and cooling problem associated with Joule dissipation in MHD flows [5].

In this paper, we investigate the stability problem of an electrically conducting fluid in a rectangular cross-section duct under a steady and uniform transverse magnetic field. The walls of the duct considered here are assumed to be perfectly electrically conducting, which is a reasonable approximation for the ducts in blankets for future nuclear fusion reactor [3]. The magnetic Reynolds number is low in the most industrial and laboratory MHD flows, thus the perturbation of the magnetic field is neglected in comparison with the imposed field and induced electric currents adjust instantaneously to the change of the velocity field [1].

In the presence of a strong transverse magnetic field, the flow in rectangular ducts with conducting walls becomes highly anisotropic as the strength of the magnetic field is increased. The MHD duct flow considered here develops with almost flat core and sharp boundary layers at the walls. There are two types of boundary layers formed depending on the orientation of the walls to the field. The Hartmann layer is located at the wall perpendicular to the magnetic field and its thickness scales as *Ha*^−1^ [6]. At the wall parallel to the magnetic field located is the Shercliff layer, also called sidewall layer, with the thickness scaling as *Ha*^−1/2^ [7]. Note that jets are developed at strong magnetic field, and the mean velocity profile is different from the case with insulating walls, in which neither jets nor inflection points exist in the mean flow. The appearance of jets and formation of inflection points in the flow field, indicates the flow maybe highly unstable, similar to Hunt’s flow [8], where strong jets are developed at a relatively weak magnetic field with a pair of conducting walls and another pair of insulating walls.

The understanding of the mechanism of instability and transition in MHD flows is almost exclusively limited to Hartmann layer rather than sidewall layer [9–12]. These studies, however, are mainly of academic significance as the sidewalls always affect the duct flow in a major way, even in the pure hydrodynamic cases. Recently, Lingwood and Alboussière [9] performed linear stability analysis of Hartmann layer, and a critical Reynolds number was determined to be 48250*Ha*, which is two orders of magnitude higher than observation in experimental studies. The onset of turbulence was only around 225*Ha* in straight duct flow [13] and 380*Ha* in annular duct flow [14], respectively. This discrepancy is also observed in other shear flows, e. g. classical channel flow and duct flow, where transition takes place at much lower value than the predicted threshold based on normal mode stability analysis. Recent developments of nonmodal stability theory [15] revealed that, even though every eigenmode decays eventually considered in normal mode theory, a combination of them, the non-normal mode can experience transient algebraic growth with the ‘lift-up’ effect [16]. Nonlinear effect could participate after a large amplification of the perturbation and the mean flow is modulated thus becomes unstable to three dimensional perturbations. The strongest amplification is normally provided by streamwise vortices, the so-called optimal mode, in hydrodynamic shear flows [17, 18]. Nonmodal stability analysis in the Hartmann flow reported similar structures in the form of streamwise vortices, which are confined in the Hartmann layers [10–12]. Based on these ideas, Krasnov et al. [19] performed direct numerical simulation (DNS) on MHD channel flow to explore a two-step transition scenario including the evolution of streamwise vortices into streaks and breakdown of streaks to subsequent transition. They found the critical local Reynolds number *R*_*c*_ was between 350*Ha* and 400*Ha*, which fits well with the experiment results [14].

However, the instability and transition behavior in the MHD channel flow can’t be extrapolated straightforward to the MHD duct flow. Firstly, the reasons are the corner interaction between the Hartmann layer and the sidewall layer and the limiting effect of the walls in duct flows. Secondly, the structure in sidewall layer is essentially three dimensional. The conductivity of the duct walls also plays an important role in the stability issue of MHD flows [20, 21]. In the limit of a perfectly conducting duct, which is not only theoretical but also of experimental relevance, weak jets persist at the parallel walls, i. e., the sidewalls. After performing normal mode stability analysis, Priede et al. [21] demonstrated that the flow in a square duct become unstable at *Ha* = 9.6. In particular, they have shown that for high value of *Ha*, Instability occurred in the form of strongly non-uniform vortices aligned with the magnetic field. In the conclusion, they suspected that the flow instability may be supercritical rather than sub-critical, due to the inviscid nature of the instability caused by the inflection points and jets. As a complementary and comparison to the above study, the present investigation is precisely to analyze the temporal growth and spatial structures of perturbations based on nonmodel stability theory. We have investigated the case of a square cross-section duct with *Re* = 5000 in a preliminary study [22], and find that the mean flow is stable at either large or small Hartmann number, but unstable at a moderate value *Ha* = 30. In the present study, the work is extended by taking account of the variation on aspect ratio *r* (width to height) and Reynolds number *Re*.

The paper is organized as follows. The problem is described in Sec. 2 below. In Sec. 3, we present the numerical results of stability analysis on MHD duct flows with different sizes. The paper is concluded in Sec. 4.

## 2 Formulation of the problem

**Fig 1:**
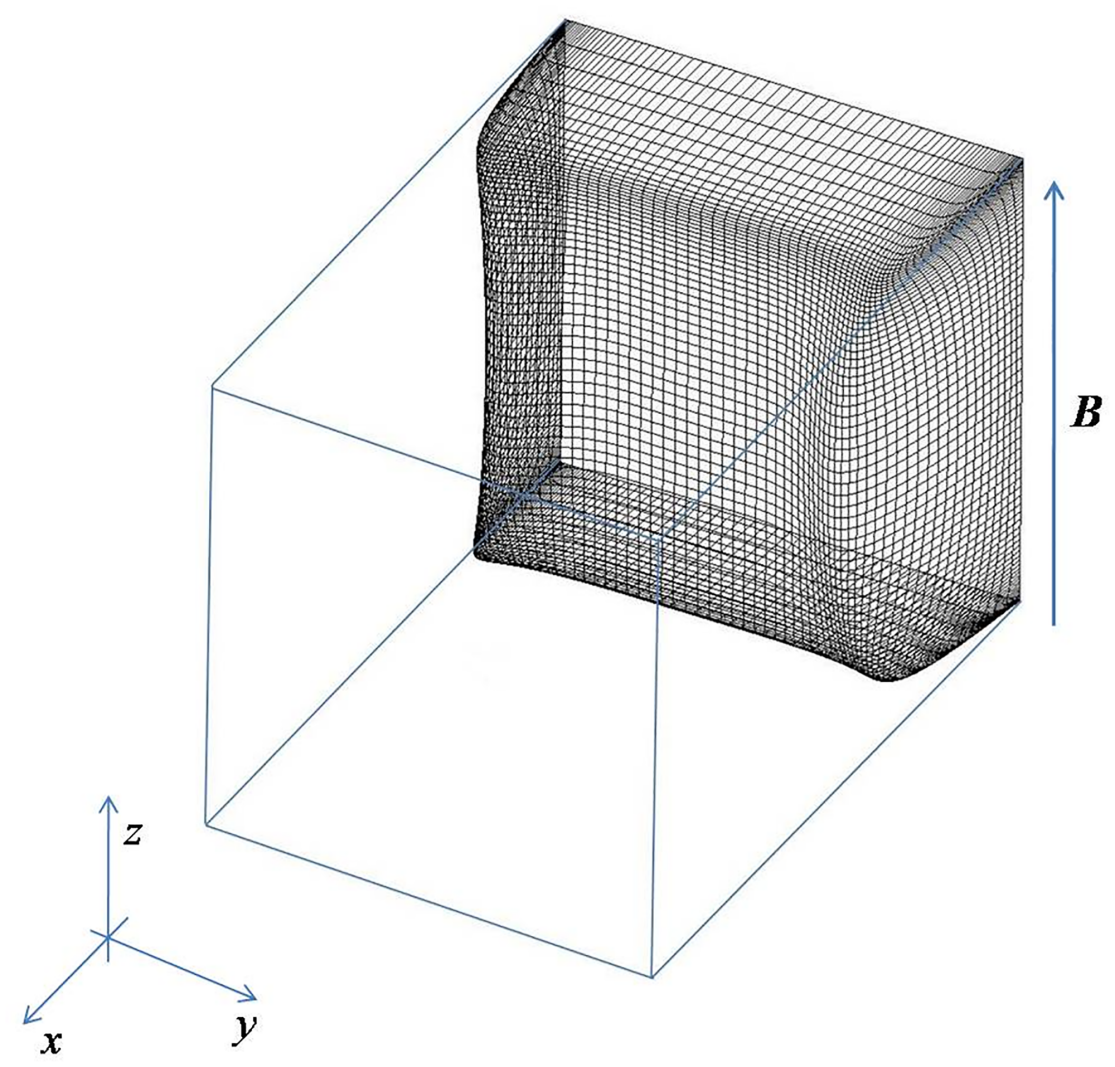
Geometry of the duct flow under magnetic field. The arrow indicates the orientation of the imposed magnetic field.

We consider the flow of an incompressible conducting liquid in a rectangular duct driven by a constant pressure gradient under a homogeneous transverse magnetic field. The walls of the duct are assumed to be perfectly electrically conducting. The magnetic field is uniform, static and aligning along the height of the duct, which is kept constant and the width of the duct is varied, as shown in Fig 1. In this study, the Cartesian coordinates is employed and the axis *x*, *y*, *z* denotes the streamwise, spanwise and cross-flow direction, respectively.

In the limit of a low magnetic Reynolds number, this problem can be described by the nondimenisonal Navier-Stokes equations for the velocity **u** and the electric potential *ϕ* as follows

$$\begin{array}{c} \frac{\partial u}{\partial t}+\left(u\cdot \nabla \right)u=-\nabla p+\frac{1}{Re}\nabla^{2}u+\frac{Ha^{2}}{Re}(-\nabla \phi \times e_{z}+(u\times e_{z})\times e_{z}), \end{array}$$ (1)

$$\begin{array}{c} \nabla \cdot u=0, \end{array}$$ (2)

$$\begin{array}{c} \nabla^{2}\phi =\nabla \cdot (u\times e_{z}), \end{array}$$ (3)

$$\begin{array}{c} u=v=w=\phi =0\;\text{at}\;z=\pm 1, \end{array}$$ (4)

$$\begin{array}{c} u=v=w=\phi =0\;\text{at}\;y=\pm r, \end{array}$$ (5)

where *p* is the pressure of the fluid, **e**_*z*_ ≡ (0, 0, 1) is the unit vector of the applied magnetic field, and *u*, *v*, *w* denotes the streamwise, spanwise and wall normal velocity component, respectively. The Ohm’s law is employed to obtain the induced electric current density

$$\begin{array}{c} j=-\nabla \phi +u\times e_{z}. \end{array}$$ (6)

For non-dimensionalization, the maximum value of laminar base flow *U*, half height of duct *L*, *L*/*U*, *ρU*^2^, *B*_0_ and *LUB*_0_ have been taken as the characteristic velocity, length, time, pressure, magnetic field and electric potential, respectively. This problem is related with two non-dimensional parameters: the Reynolds number

$$\begin{array}{c} \text{Re}\equiv UL/\nu \end{array}$$ (7)

and the Hartmann number

$$\begin{array}{c} Ha=L/\delta ,\;\delta ={\left(\rho \nu /\sigma B_{0}^{2}\right)}^{1/2}, \end{array}$$ (8)

where *δ* denotes the thickness of the Hartmann layer. The local Reynolds number is then defined as *R* = *Uδ*/*ν* = *Re*/*Ha*. The boundary condition at the walls for the velocity is no slip condition, and that for electric potential is *ϕ* = 0 for conducting walls, and $\frac{\partial \phi }{\partial n}=0$ for insulating walls, respectively.

For the analysis of arbitrary perturbations, the governing Eqs (1–3) are linearized around the Hartmann duct flow **U**(*y*, *z*, *t*). The linear perturbations then take the form

$$\begin{array}{c} u_{p}\left(x,y,z,t\right)=\frac{1}{2}[\hat{u}\left(y,z,t\right)\exp\left(i\alpha x\right)+c.c.], \end{array}$$ (9)

where *α* denotes the streamwise wavenumber, *c*.*c*. stands for complex conjugation, and the complex vector $\hat{u}$ has the components $\hat{u}=\left(\hat{u},\hat{v},\hat{w}\right)$.

In order to qualify the amplification of perturbations, it is customary to define an energy norm as follows,

$$\begin{array}{c} E\left(t\right)\equiv \int \left(\hat{u}{\hat{u}}^{*}+\hat{v}{\hat{v}}^{*}+\hat{w}{\hat{w}}^{*}\right)dydz, \end{array}$$ (10)

Where the superscript star denotes complex conjugation. We then evaluate the amplification factor *G* as the ratio *E*(*t*)/*E*(0). This quantity need to be maximized over all possible initial perturbation profiles in [0, *τ*] to get the maximum value with a specific wavenumber *α*. This optimization is constraint with two limits: (i) the disturbance must satisfy the linear governing equations as well as the boundary conditions in [0, *τ*]; (ii) the disturbance energy at time *t* = 0 is conveniently set to equal to unity for simplicity. We incorporate the constraints into the energy functional with the assistance of Lagrangian multipliers [23], which are actually the adjoint field of variables. The adjoint field variables satisfy linear adjoint equations, which are in a similar form of Eqs (1–3).

Then the amplification of perturbation *G* is obtained by an iterative procedure, which is illustrated below

$$\begin{array}{c} \begin{array}{ccc} \hat{u}\left(y,z,0\right) & \overset{Direct}{⟶} & \hat{u}\left(y,z,\tau \right)\\ ↑ & & ↓\\ \tilde{u}\left(y,z,0\right) & \overset{Adjoint}{⟵} & \tilde{u}\left(y,z,\tau \right). \end{array} \end{array}$$

Any given initial condition is firstly propagated forward in time by solving the direct problem, followed by an backward propagation of the adjoint field, in which an initial condition is derived from the direct field at time *t* = *τ*. After one integration, an updated initial condition for the next iterative step is available. Convergence is reached when the initial condition satisfies an appropriately chosen criterion from one iterative step to the next one. In the present study, we choose |(*G*_*n*_ − *G*_*n*−1_)/*G*_*n*−1_| < 1*e* − 7, where *G*_*n*_ is the energy amplification factor at the *n*th iteration step, and *G*_*n* − 1_ is the one at the (*n* − 1)th iteration step. The maximum energy amplification is eventually obtained by iterating the converged initial condition forward in time and computing the ratio *E*(*τ*)/*E*(0).

The derivation of the adjoint equations and the iteration procedure are analogous to that for the duct flow with insulating walls presented in Krasnov et al. [24]. The direct and adjoint governing equations are solved numerically with a modified finite difference code used in our prior studies [22, 24, 25]. More details can be found in the references listed above.

## 3 Results and discussions

### 3.1 Base flow

For stability analysis, the equations for a MHD laminar base flow is firstly solved by the finite difference method [22, 24, 25]. The base flow is steady, purely streamwise and independent of the streamwise coordinate. The dimensionless governing equations with the boundary conditions for the streamwise velocity component *U*_*B*_(*y*, *z*) and the corresponding electric potential *ϕ*_*B*_(*y*, *z*) are

$$\begin{array}{c} \frac{\partial^{2}U_{B}}{\partial y^{2}}+\frac{\partial^{2}U_{B}}{\partial z^{2}}=Ha^{2}(\frac{\partial \phi_{B}}{\partial y}+U_{B})-1, \end{array}$$ (11)

$$\begin{array}{c} \frac{\partial^{2}\phi_{B}}{\partial y^{2}}+\frac{\partial^{2}\phi_{B}}{\partial z^{2}}=-\frac{\partial U_{B}}{\partial y}, \end{array}$$ (12)

$$\begin{array}{c} U_{B}=\phi_{B}=0\;\text{at}\;z=\pm 1, \end{array}$$ (13)

$$\begin{array}{c} U_{B}=\phi_{B}=0\;\text{at}\;y=\pm r. \end{array}$$ (14)

The computational solution has been verified for all aspect ratios *r* in the range [1, 9], and different Hartmann numbers *Ha* in [0, 50] with *Re* = 3000, 5000 and 7000, through a comparison with the analytical solution obtained by Hunt et al. [8].

**Fig 2:**
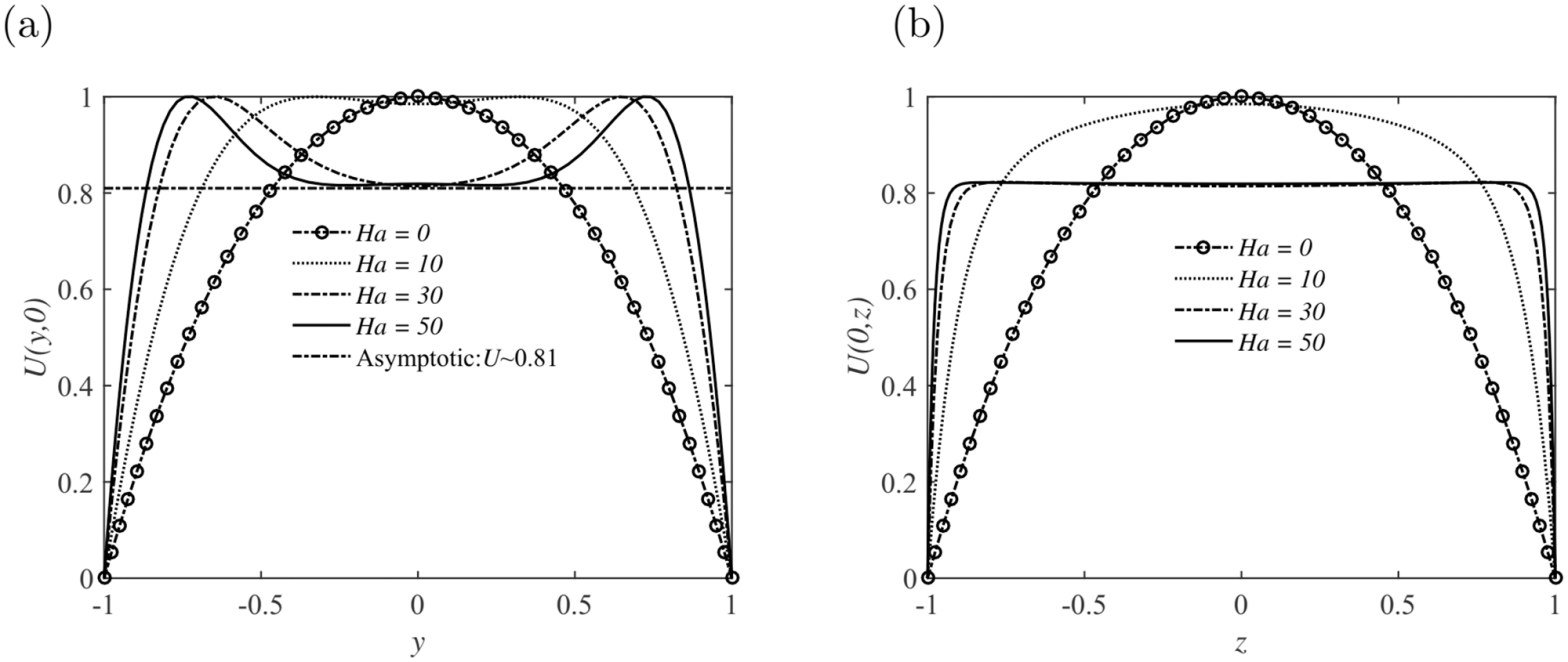
Basic velocity profiles for different Hartmann numbers *Ha* in the square cross-section duct. (a) Velocity profiles *U*(*y*, *z* = 0) within the sidewall layers; (b) Velocity profiles *U*(*y* = 0, *z*) in the central cross-section parallel to the sidewall of the duct.

**Fig 3:**
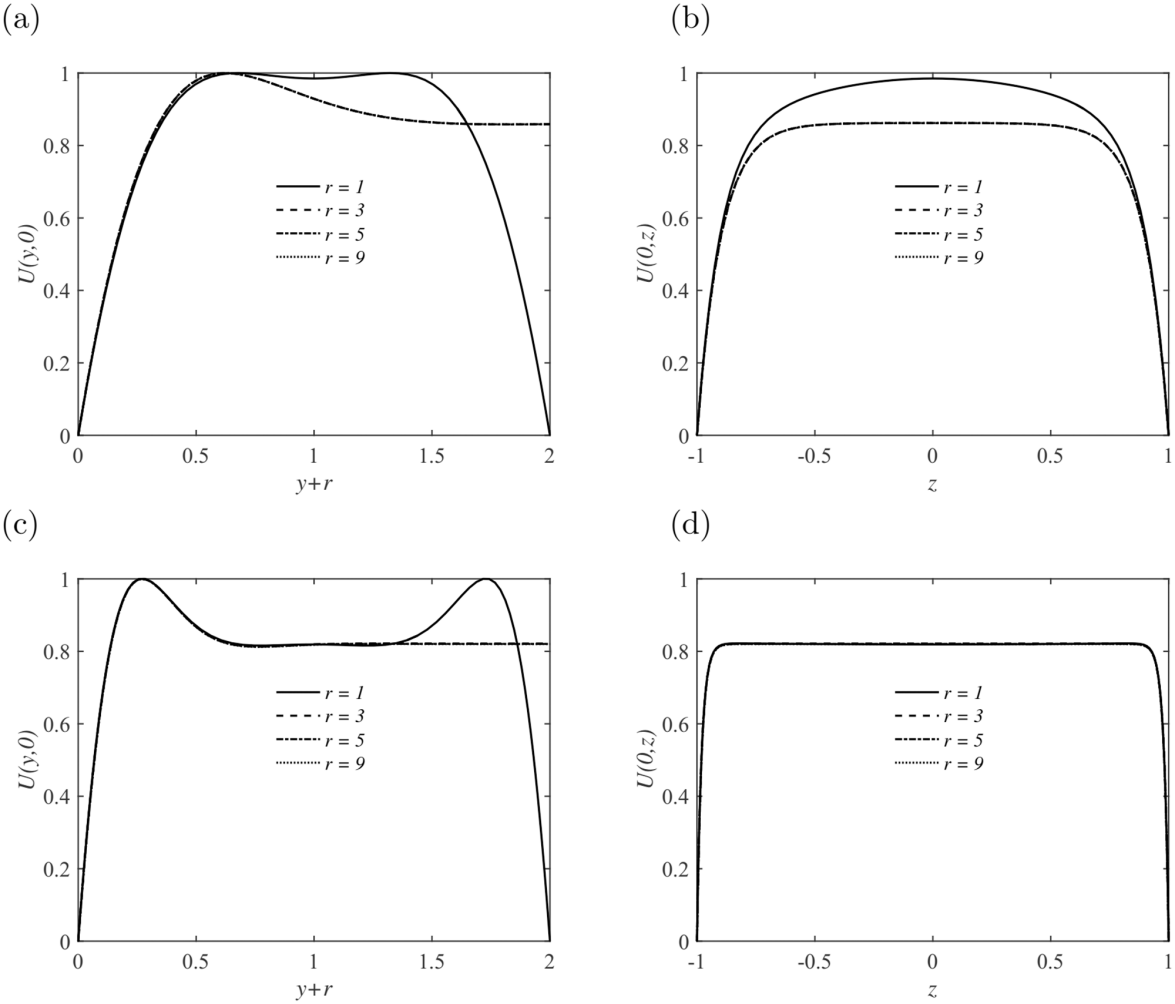
Basic velocity profiles in ducts with different aspect ratio *r* at *Ha* = 10 (a, b) and *Ha* = 50 (c, d). Velocity profiles *U*(*y* + *r*, *z* = 0) within the sidewall layers (a, c); Velocity profiles *U*(*y* = 0, *z*) in the central cross-section parallel to the sidewall of the duct (b, d).

As shown in Fig 2, the laminar velocity profile in the duct is reproduced with *Re* = 3000 and *Ha* = 0. A strong magnetic field is then imposed along the direction of the height of the duct, as shown in Fig 1, the core flow becomes flat with weak jets developing in the sidewall layers. The base velocity profiles with different *Ha* in the *y* and *z* axis are also demonstrated in Fig 2, respectively. The mean velocity profile becomes more flat and the thickness of the boundary layer becomes thinner with the growing of *Ha*. Note that, the structure of the Schercliff layer changes with *Ha* but is practically insensitive to the variation of aspect ratio *r*, as shown in Fig 3.

Stability analysis on the perturbations developing in the duct also indicates the insensitiveness of perturbation structures to *r*. The maximum value of the base flow velocity has been taken as the characteristic velocity for nondimensionalization, as suggested in the reference [21]. Given a sufficiently large *Ha*, an asymptotic limit will be reached for the base velocity in the middle cross section as *U* ≈ 0.81, as shown in Figs 2(b) and 3(d), which is confirmed by our numerical solutions.

The base flow is symmetric with respect to *y* = 0 and *z* = 0 axis, thus the perturbations with different parties in *y* and *z* can be decoupled from each other. We classify four independent modes as (*o*, *o*), (*o*, *e*), (*e*, *o*) and (*e*, *e*) according to the *y* and *z* symmetry of $\hat{w}$, respectively. This classification corresponds to the Mode I, II, III and IV used in references [26, 27].

### 3.2 Results of nonmodal stability analysis

**Fig 4:**
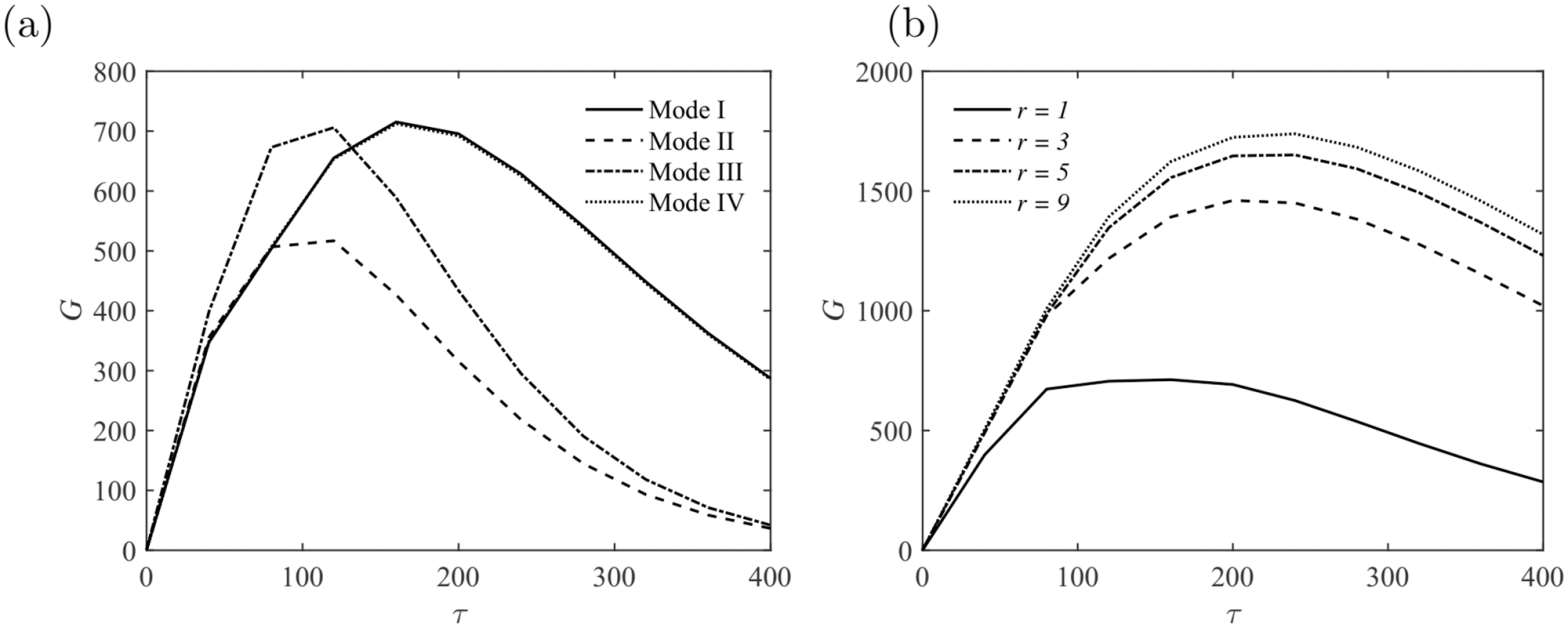
(a) Energy amplification of perturbations *G* with different modes at *Re* = 3000 and *Ha* = 0; (b) Energy amplification of perturbations *G* in the duct with different aspect ratios *r* at *Re* = 3000 and *Ha* = 0.

In this section, a lower Reynolds number *Re* = 3000 with different Hartmann number *Ha* = 0, 10, 20 and 30 is firstly investigated. The effect of the aspect ratio *r* = 1, 3, 5 and 9 is also taken into consideration. The amplification factor *G* and the corresponding wavenumber *α* are presented in Fig 4 with *Re* = 3000 and *Ha* = 0. As the aspect ratio *r* is increased, the amplification factor *G* is getting closer to the limit *G*_*max*_ ≈ 1800, which corresponds to *r* → ∞, i. e., the channel case. The discontinuities in the slope of the global amplification curves (solid line in Fig 4(b)) is an effect of the changes of optimal mode at different *τ*. The optimal mode is in the form of Mode III at short time *τ* ≤ 130, and then taken over by Mode I at *τ* ≥ 130 in the square duct *r* = 1, as illustrated in Fig 4(a). The global optimization is found to take the form of Mode I, with *G*_*max*_ ≈ 712 and *τ*_*opt*_ ≈ 160. Both peaks of perturbations in the form of Mode III and Mode I provide almost identical level of amplification. For MHD flows in the larger size of duct with *r* ≥ 3, the peak at large time *τ* provided by Mode I is more pronounced. This finding is consistent with previous studies on non-MHD duct flow and channel flow [18, 28].

**Fig 5:**
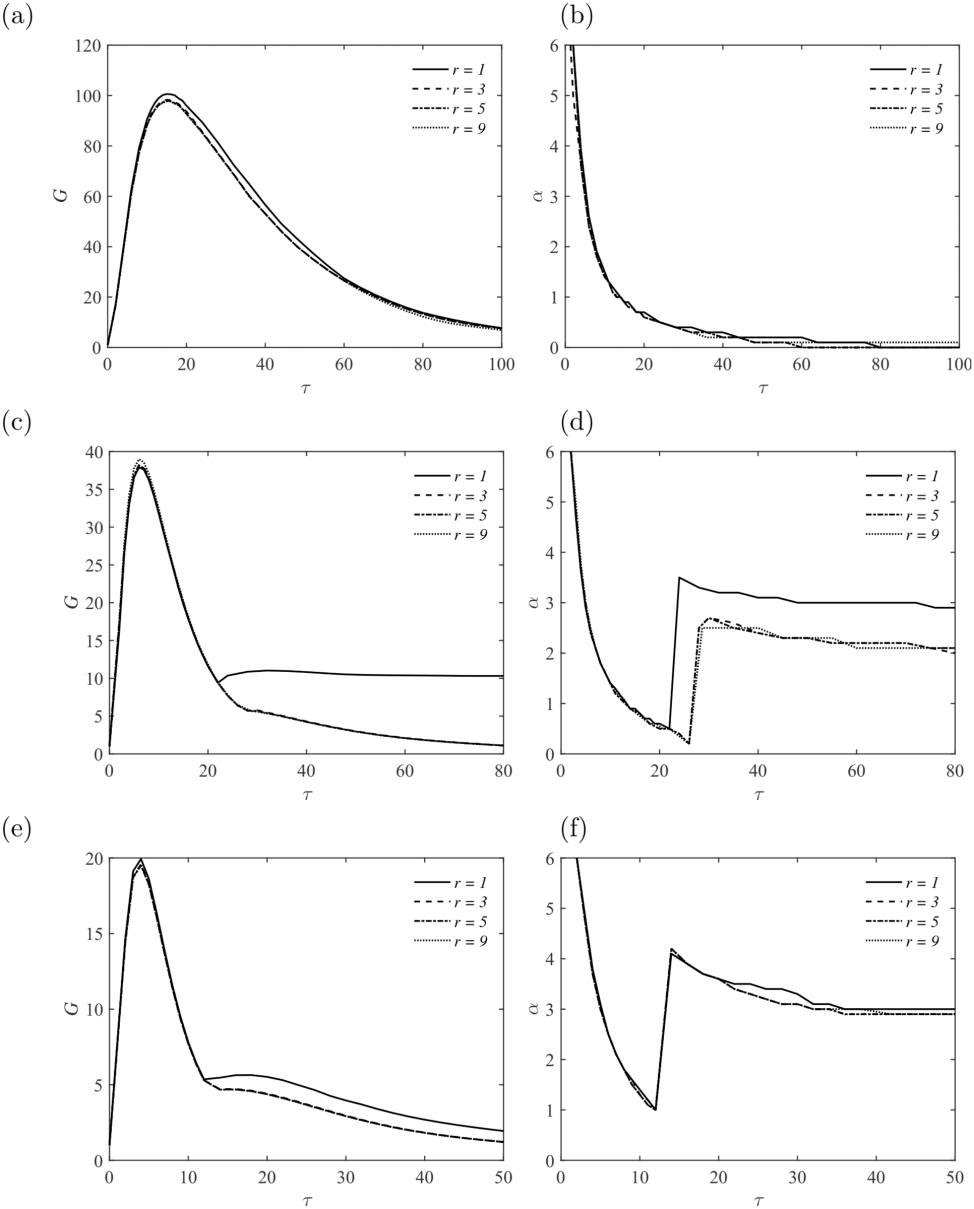
Energy amplification of perturbations *G* and the corresponding streamwise wavenumber *α* as a function of *τ* at *Re* = 3000. (a, b) *Ha* = 10; (c, d) *Ha* = 20; (e, f) *Ha* = 30.

At non-zero magnetic field, perturbations experience strong Joule dissipation and suppression than non-MHD cases, thus the amplification will be reduced to some extent depending on the strength of the imposed magnetic field, i. e., the Hartmann number *Ha*. For all Hartmann numbers considered at *Re* = 3000, perturbations with different streamwise wavenumber *α* grow transiently at small *τ* and decay eventually at large *τ*, as shown in Fig 5. There is no observation of exponential growth of perturbations, which may be contributed to the lower Reynolds number considered here. Although jets are developed in the flow field (see Figs 2 and 3), they are too weak to render the flow unstable to three dimensional perturbations, in the presence of a strong magnetic field.

The most striking feature is the irrelevance of the amplification factor *G* to the aspect ratio *r*, especially at small *τ*, as shown in Fig 5. It becomes apparent that, c. f. Fig 3, the sidewall is far away from each other when *r* ≥ 3, which leads to the decouple of the two sidewall layers. This is also the reason of the discrepancy of *G* curves with *r* = 1 and *r* ≥ 3 at large *τ*.

**Fig 6:**
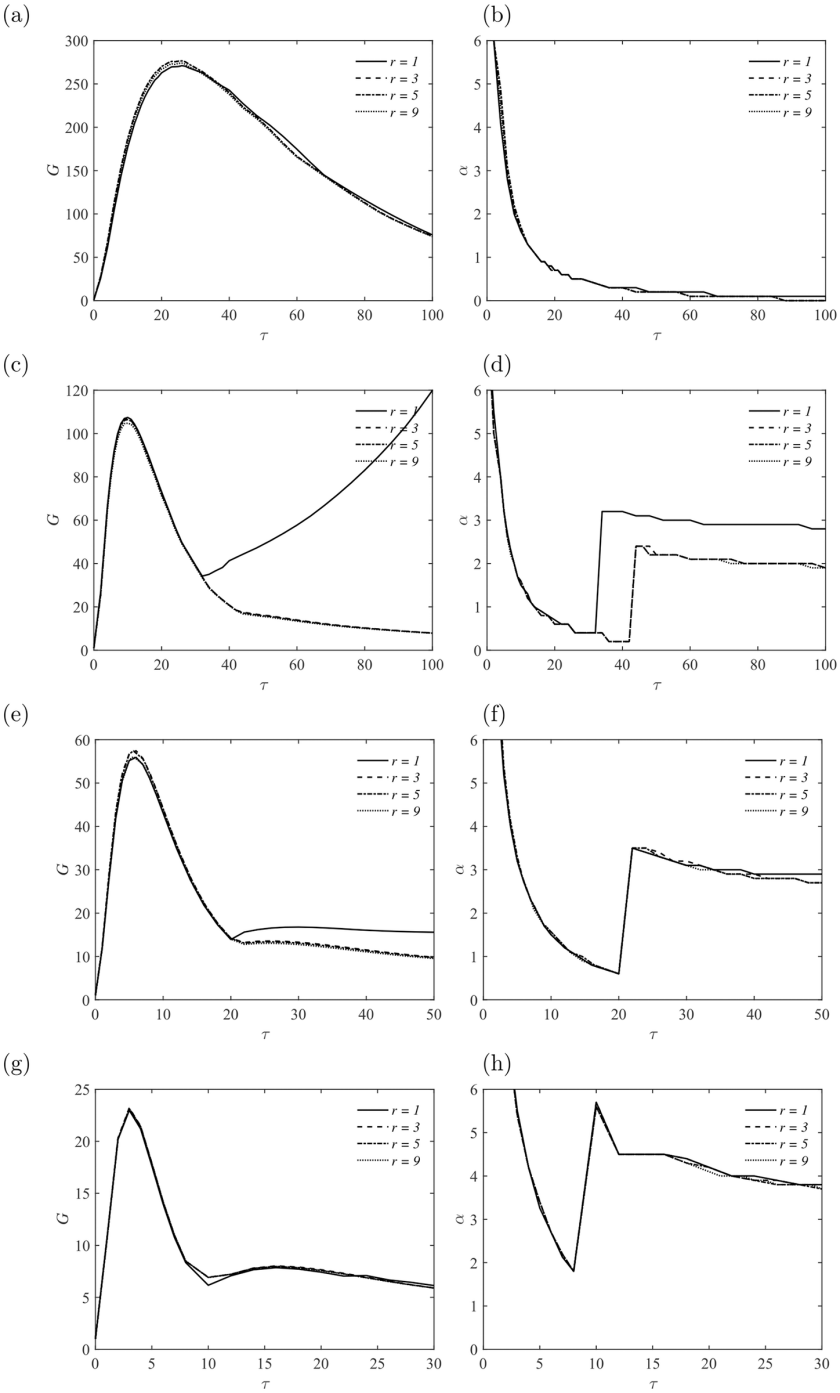
Energy amplification of perturbations *G* and the corresponding streamwise wavenumber *α* as a fuction of *τ* at *Re* = 5000. (a, b) *Ha* = 10; (c, d) *Ha* = 20; (e, f) *Ha* = 30; (g, h) *Ha* = 50.

Similar trends of the growth of perturbations are observed for *Re* = 5000 with *Ha* = 10, 20, 30 and 50. At higher Reynolds number, the amplification factor *G* becomes relatively larger and the corresponding optimal mode is obtained at larger *τ*, while the optimal wavenumber keeps nearly unchanged, as illustrated in Fig 6. At *Ha* = 10, the global maximum value of amplification *G* is obtained with perturbations in the form of Mode I at the optimal time *τ* ≈ 30. The *G* and *α* curves for different sizes of duct are nearly discernible, which implies similar structures exist in the flow field. A detail explanation is described in the later section.

Exponential growth of perturbations is captured only in the square duct (*r* = 1) at *Ha* = 20. This feature is absent in the other duct flows with aspect ratio *r* ≥ 3. As demonstrated in Fig 3, jets are formed in the sidewall layers and the structure is independent of the aspect ratio *r*. Hence, there may still be residual interactions between the two sidewall layers, especially when they are close, e. g., *r* = 1, and perturbations may extract energy efficiently from the jets flow and grow rapidly [5, 20]. As the aspect ratio *r* is increased, the interaction becomes too weak to give chance to the growth of perturbations, meanwhile, the imposed magnetic filed tends to suppress fluctuations along the field line. When *Ha* is increased from 20 to 30, a lower exponential growth rate of the most unstable perturbation in the form of Mode I is obtained. The corresponding wavenumber *α* is around 3. Again, there is no exponential growth of perturbation within the ducts *r* ≥ 3 due to the decouple of jets in the sidewall layers and Joule dissipation effect in the presence of a strong magnetic field.

Finally, an even strong magnetic field with *Ha* = 50 is imposed to investigate the MHD flow stability in different sizes of duct. The effect of the magnetic field is twofold, as mentioned above. On the one side, core flow becomes flat and jets are formed in the sidewall layers, thus the flow becomes more unstable. On the other side, all perturbations are supposed to be suppressed by the imposed magnetic field, thus the mean flow should be more stable. At *Ha* = 50 with *Re* = 5000, the latter mechanism is dominated and no exponential growth of perturbations is observed even in the square duct. However, there is still a second peak, corresponding to perturbations with a streamwise wavenumber *α* ≈ 4, which eventually decay due to the strong Joule dissipation and viscous dissipation.

**Fig 7:**
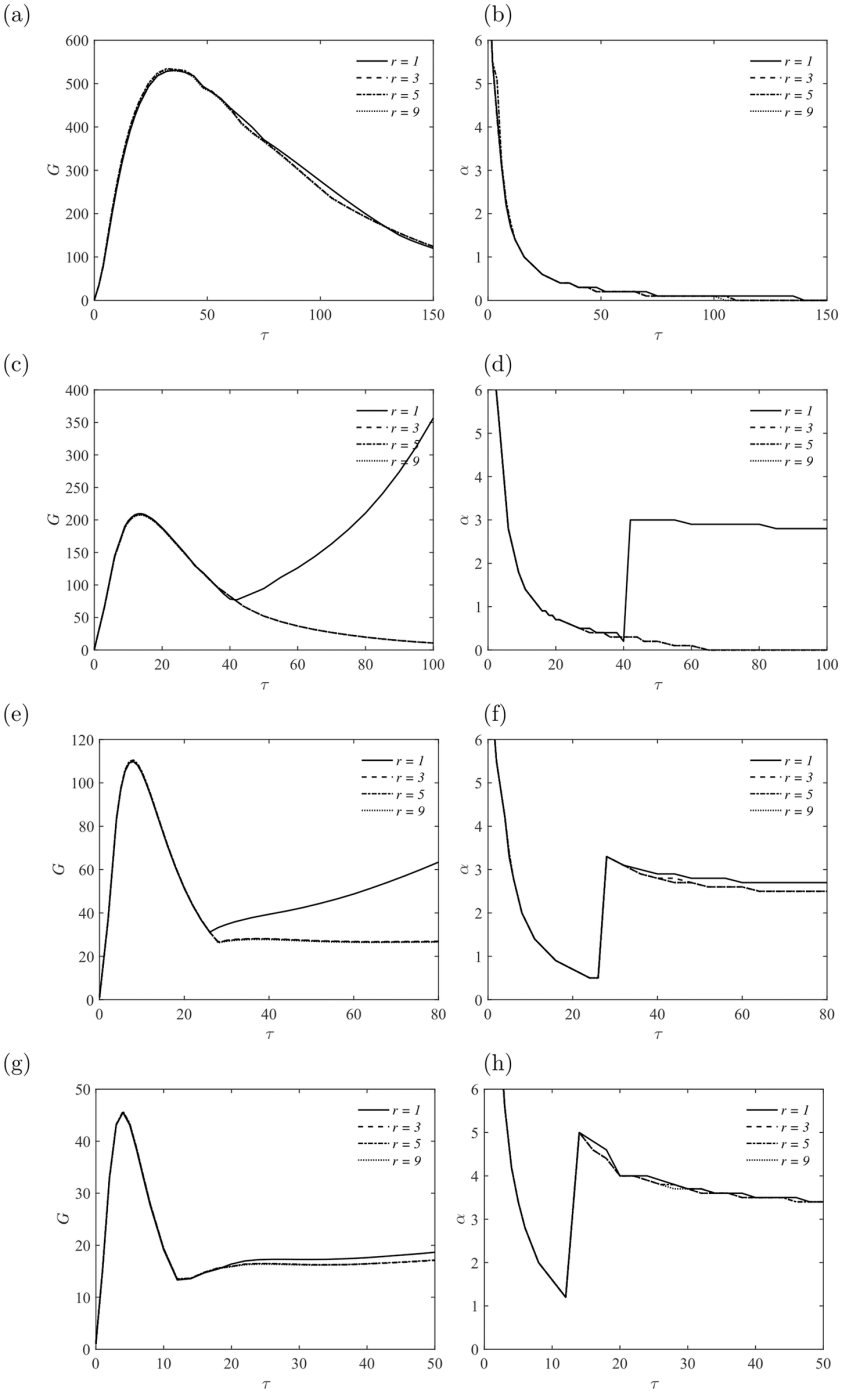
Energy amplification of perturbations *G* and the corresponding streamwise wavenumber *α* as a fuction of *τ* at *Re* = 7000. (a, b) *Ha* = 10; (c, d) *Ha* = 20; (e, f) *Ha* = 30; (g, h) *Ha* = 50.

Similar to the results at *Re* = 5000, exponential growth of perturbations is observed at intermediate Hartmann number (20 ≤ *Ha* ≤ 50) and *Re* = 7000, as demonstrated in Fig 7. Exponential growth of perturbations is observed at *Ha* = 50 with aspect ratio *r* ≥ 3, in which the interaction between the two sidewall layers becomes stronger at higher *Re* and *Ha*, and may contribute to the growth of perturbations in special forms, i. e., the most unstable modes.

### 3.3 Profiles of optimal perturbations

**Fig 8:**
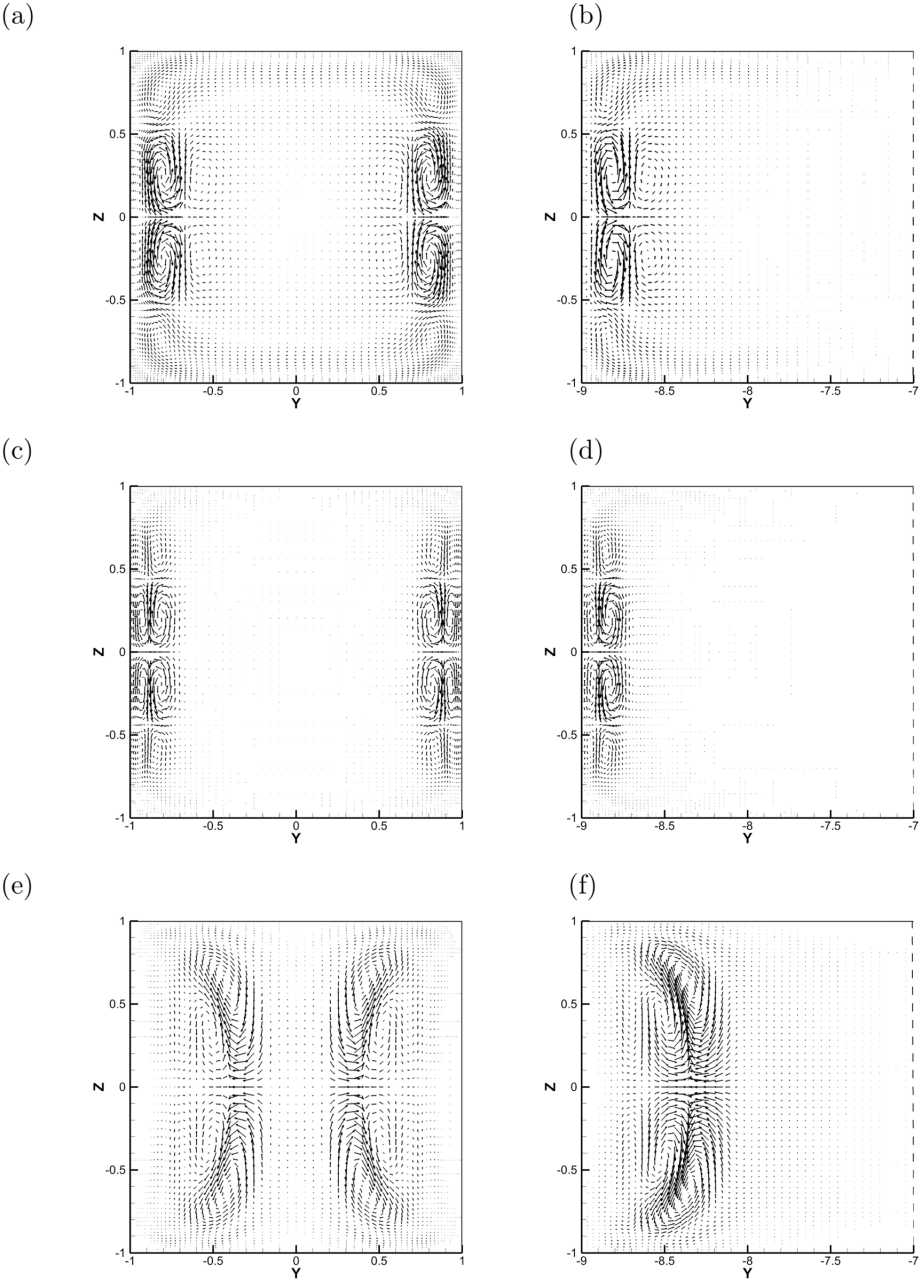
The spatial structures of optimal perturbations at initial with *Re* = 3000 and *r* = 1 (left column) and *r* = 9 (right column). (a) *τ* = 15, *α* = 0.9, and *Ha* = 10; (b) *τ* = 15, *α* = 0.9, and *Ha* = 10; (c) *τ* = 6, *α* = 2.4, and *Ha* = 20; (d) *τ* = 6, *α* = 2.4, and *Ha* = 20; (e) *τ* = 30, *α* = 3.2, and *Ha* = 20; (f) *τ* = 30, *α* = 2.7, and *Ha* = 20.

**Fig 9:**
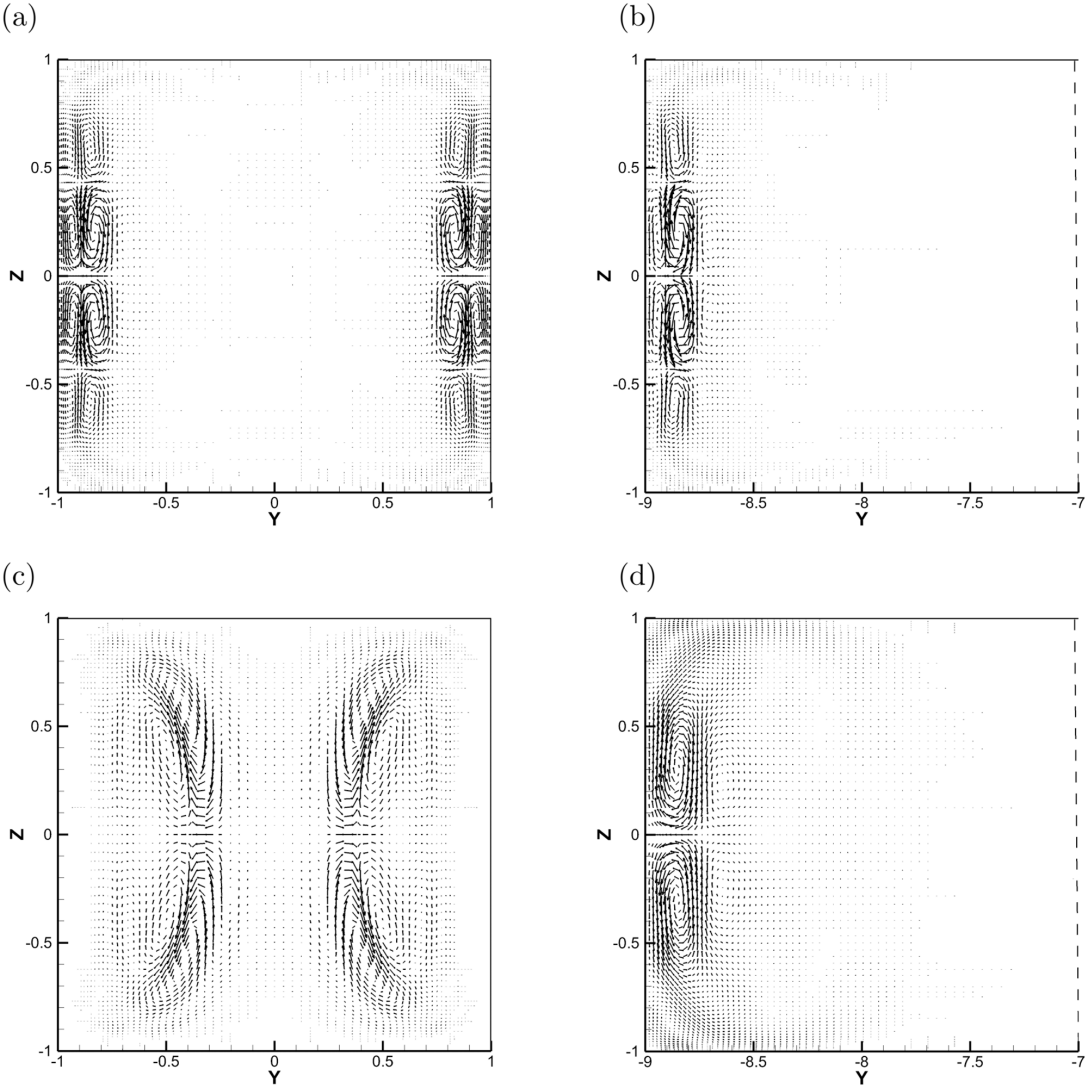
The spatial structures of optimal perturbations at initial with *Re* = 7000, *Ha* = 20 and *r* = 1 (left column) and *r* = 9 (right column). (a) *τ* = 13, *α* = 1.2; (b) *τ* = 13, *α* = 1.2; (c) *τ* = 50, *α* = 3.0; (d) *τ* = 50, *α* = 0.2.

**Fig 10:**
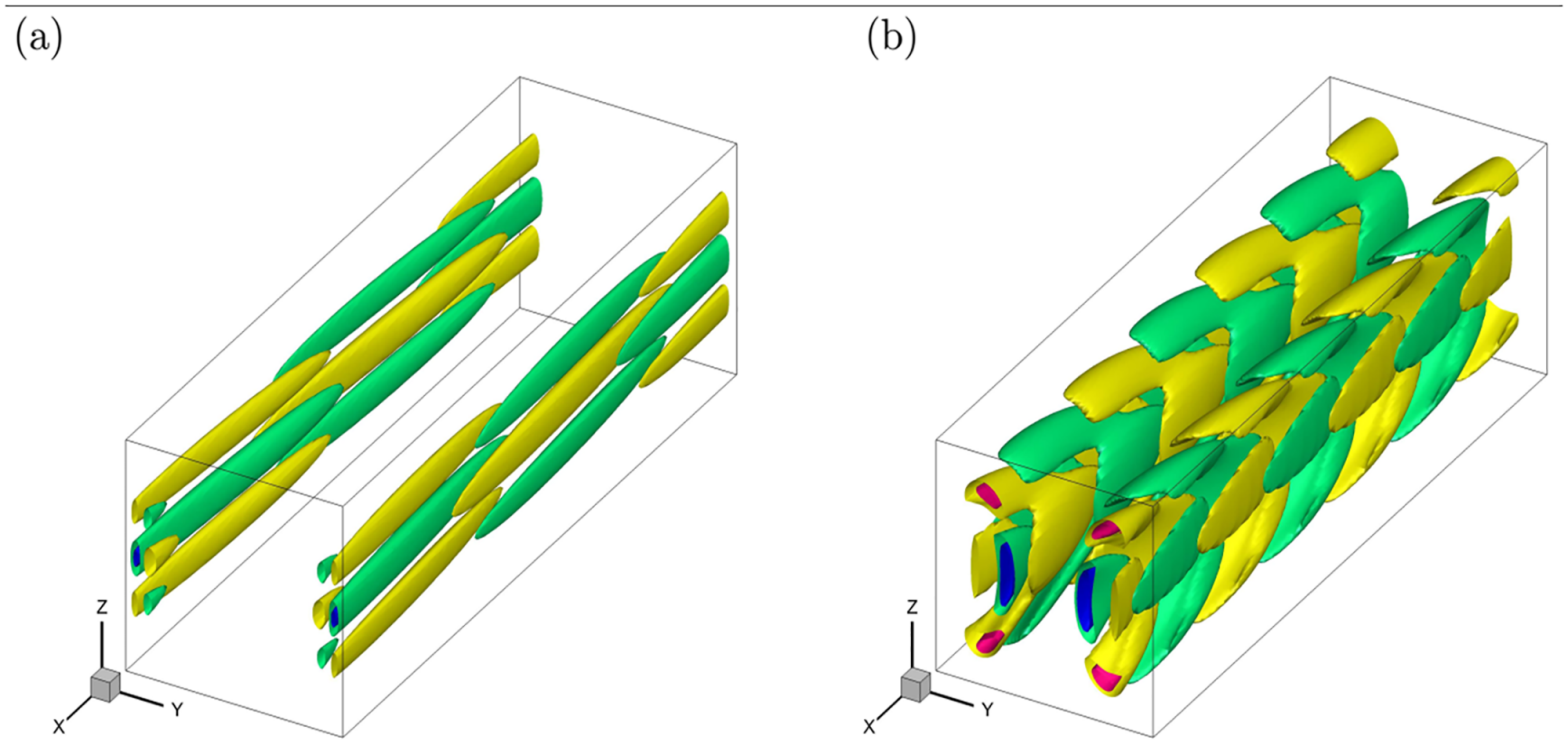
The optimal perturbation at different final time *τ* for *Re* = 7000 and *Ha* = 20 in the square cross-section duct. (a) *τ* = 13, *α* = 1.2; (b) *τ* = 50, *α* = 3.0. The iso-surfaces correspond to the ±0.45 and ±0.90 of the maximum magnitude of the streamwise velocity component.

The spatial structure of the optimal perturbations is demonstrated in Figs 8–10 for non-zero magnetic field. The optimal perturbations for the duct with *r* = 9 shown in the right column of Fig 8 have structures similar to that with *r* = 1 in the left column at *Re* = 3000. At small *τ*, the growth of optimal perturbations is transient and they are localized within the Schercliff layers at initial (Fig 8(a)–8(d)). By contrast, at large *τ* in some cases, the optimal perturbations grow exponentially and are located in the jets zone at initial (Fig 8(e) and 8(f)). For both perturbations, the localization of perturbations indicates that increasing *r*, i. e. extending the width of the duct, does not affect their development. This invariance with aspect ratio *r* is also demonstrated in Fig 4(a) and 4(c), in which the base velocity profiles with different *r* convergent near the sidewalls. Similar structures of initial optimal perturbations are observed in Fig 9 with *Re* = 7000. Note that, the optimal streamwise wavenumber *α* in Fig 9(d) is close to zero, *α* = 0.2, which is different from other cases. The spatial profile is more like the one found in pure hydrodynamic cases, i. e., the streamwise vortices [17, 18].

The final state of the optimal perturbations are shown in Fig 10, and the structures are inhomogeneous in the *x* direction and form complex overlapping patterns within the Schercliff layers (Fig 10(a)) or in the fully developed jets zone (Fig 10(b)). The distribution of perturbation energy among the velocity components varies with *Ha*. In the initial state, *u*^2^, *v*^2^, *w*^2^ contain 3.63%, 9.56%, 86.81%, respectively, of the total kinetic energy with *α* = 1.2 (Fig 9(a)) and 62.30%, 6.56%, 31.14% with *α* = 3.0 (Fig 9(c)). In the final state investigated, the distribution changes to 96.56%, 0.43%, 3.01% with *α* = 1.2 (Fig 10(a)) and to 59.59%, 6.77%, 33.64% with *α* = 3.0 (Fig 10(b)). If one identifies streaks with the dominance of the streamwise velocity component, we conclude that the optimal perturbations is dominated by the streak-like structures and the mechanism of transient growth of perturbations is ‘lift-up’ effect [16] in Fig 10(a). As for Fig 10(b), the percentage of kinetic energy in each component is unchanged and this may be related with the Orr mechanism [29] of exponential growth of perturbations.

## 4 Conclusions

The stability of MHD duct flow with different size is investigated in this study with the assumption of electrically perfectly conducting walls. A static and homogeneous magnetic field is imposed along the height of the duct, which is kept constant while the width of the duct is varied. Three Reynolds numbers are considered with different Hartmann numbers varied from 0 to 50, and the aspect ratio *r* (width to height) of the duct is changed in the range [1, 9]. The stability problem is solved with nonmodal stability analysis method, and the growth of perturbations, as well as the spatial structures are obtained.

The MHD duct flow is found to be unstable at larger Reynolds number, but with moderate Hartmann number. At lower Hartmann number, perturbations tend to be suppressed by the imposed magnetic field and the growth of perturbations turns out to be transient. For MHD duct flows at higher Hartmann number, although there are jets and inflection points formed in the mean flow, the growth of perturbations is also transient rather than exponential due to the strong Joule dissipation and viscous dissipation. The fundamental mechanism of transient growth appears to be the hydrodynamic ‘lift-up’ effect. This conclusion is based on the relative magnitude of perturbation velocity component at initial and the final state of transient growth. While the fundamental mechanism of exponential growth of perturbations evolving on the jets is related with the Orr mechanism.

The computational results show that, MHD flow in larger size of duct (*r* ≥ 3) is independent of the aspect ratio *r* on account of the invariance of the basic flow within the Schercliff layers. From this perspective, the MHD flow in a square cross-section duct with conducting walls captures nearly all the relevant aspects of the behavior for larger *r*.

The conductivity of the walls plays an important role in the stability issue of MHD flows. As reported in the previous study [24], there is no appearance of jets and inflection points in the MHD duct flows with insulating walls. The perturbations grow transiently and the mean flow is relatively stable compared with the present consideration with conducting walls in the same range of parameters.

Recent simulations in Hunt’s flow [30] indicate that transition starts in the Schercliff layers as expected from the transient growth analysis. It would be interesting to analyze the transient growth of perturbations at larger *Re* and *Ha*. Although it is reasonable to believe that the key results of the present study, such as the concentration of the perturbations in Schercliff layers, are retained in a wider parameter range. Some new features may appear and need to be explored further.

## Supporting information

S1 File All the data presented within this paper is contained in the zip file. (ZIP)
